# Supplementary material for: Pyruvate Dehydrogenase Kinase 1 inhibition mediated oxidative phosphorylation enhancement in cartilage promotes osteoarthritis progression
Source: BMC Musculoskelet Disord. 2023 Jul 20;24:597. doi: 10.1186/s12891-023-06585-6 (PMC10357736; doi:10.1186/s12891-023-06585-6)
Supplement: Supplementary file 1 — Additional file 1. [file 12891_2023_6585_MOESM1_ESM.zip › MF.Gsea.1653627316242/GOMF_OXIDOREDUCTASE_ACTIVITY_ACTING_ON_THE_ALDEHYDE_OR_OXO_GROUP_OF_DONORS_NAD_OR_NADP_AS_ACCEPTOR.html]

Details for gene set GOMF\_OXIDOREDUCTASE\_ACTIVITY\_ACTING\_ON\_THE\_ALDEHYDE\_OR\_OXO\_GROUP\_OF\_DONORS\_NAD\_OR\_NADP\_AS\_ACCEPTOR[GSEA]

|  || Dataset | 55457\_collapsed\_to\_symbols.cls.cls #OA\_versus\_normal.cls.cls #OA\_versus\_normal\_repos |
| Phenotype | cls.cls#OA\_versus\_normal\_repos |
| Upregulated in class | OA |
| GeneSet | GOMF\_OXIDOREDUCTASE\_ACTIVITY\_ACTING\_ON\_THE\_ALDEHYDE\_OR\_OXO\_GROUP\_OF\_DONORS\_NAD\_OR\_NADP\_AS\_ACCEPTOR |
| Enrichment Score (ES) | 0.43402365 |
| Normalized Enrichment Score (NES) | 1.5906777 |
| Nominal p-value | 0.020408163 |
| FDR q-value | 0.14446978 |
| FWER p-Value | 0.988 |
Table: GSEA Results Summary

  

Fig 1: Enrichment plot: GOMF\_OXIDOREDUCTASE\_ACTIVITY\_ACTING\_ON\_THE\_ALDEHYDE\_OR\_OXO\_GROUP\_OF\_DONORS\_NAD\_OR\_NADP\_AS\_ACCEPTOR      
 Profile of the Running ES Score & Positions of GeneSet Members on the Rank Ordered List

  

| SYMBOL | TITLE | RANK IN GENE LIST | RANK METRIC SCORE | RUNNING ES | CORE ENRICHMENT || 1 | PDHB | pyruvate dehydrogenase E1 subunit beta [Source:HGNC Symbol;Acc:HGNC:8808] | 149 | 0.862 | 0.0758 | Yes |
| 2 | DLD | dihydrolipoamide dehydrogenase [Source:HGNC Symbol;Acc:HGNC:2898] | 565 | 0.635 | 0.1078 | Yes |
| 3 | CYP27A1 | cytochrome P450 family 27 subfamily A member 1 [Source:HGNC Symbol;Acc:HGNC:2605] | 611 | 0.619 | 0.1671 | Yes |
| 4 | HSD17B12 | hydroxysteroid 17-beta dehydrogenase 12 [Source:HGNC Symbol;Acc:HGNC:18646] | 795 | 0.570 | 0.2106 | Yes |
| 5 | ALDH2 | aldehyde dehydrogenase 2 family member [Source:HGNC Symbol;Acc:HGNC:404] | 1003 | 0.517 | 0.2469 | Yes |
| 6 | ALDH3A2 | aldehyde dehydrogenase 3 family member A2 [Source:HGNC Symbol;Acc:HGNC:403] | 1263 | 0.465 | 0.2739 | Yes |
| 7 | ALDH18A1 | aldehyde dehydrogenase 18 family member A1 [Source:HGNC Symbol;Acc:HGNC:9722] | 1402 | 0.443 | 0.3080 | Yes |
| 8 | ALDH5A1 | aldehyde dehydrogenase 5 family member A1 [Source:HGNC Symbol;Acc:HGNC:408] | 1661 | 0.405 | 0.3290 | Yes |
| 9 | ALDH1A1 | aldehyde dehydrogenase 1 family member A1 [Source:HGNC Symbol;Acc:HGNC:402] | 1737 | 0.393 | 0.3630 | Yes |
| 10 | AKR1C3 | aldo-keto reductase family 1 member C3 [Source:HGNC Symbol;Acc:HGNC:386] | 1996 | 0.361 | 0.3796 | Yes |
| 11 | ALDH7A1 | aldehyde dehydrogenase 7 family member A1 [Source:HGNC Symbol;Acc:HGNC:877] | 2158 | 0.343 | 0.4018 | Yes |
| 12 | PDHA1 | pyruvate dehydrogenase E1 subunit alpha 1 [Source:HGNC Symbol;Acc:HGNC:8806] | 2433 | 0.310 | 0.4119 | Yes |
| 13 | ALDH9A1 | aldehyde dehydrogenase 9 family member A1 [Source:HGNC Symbol;Acc:HGNC:412] | 2795 | 0.270 | 0.4113 | Yes |
| 14 | FAR2 | fatty acyl-CoA reductase 2 [Source:HGNC Symbol;Acc:HGNC:25531] | 2999 | 0.250 | 0.4208 | Yes |
| 15 | AKR1B1 | aldo-keto reductase family 1 member B [Source:HGNC Symbol;Acc:HGNC:381] | 3138 | 0.236 | 0.4340 | Yes |
| 16 | GAPDHS | "glyceraldehyde-3-phosphate dehydrogenase, spermatogenic [Source:HGNC Symbol;Acc:HGNC:24864]" | 3466 | 0.205 | 0.4294 | No |
| 17 | ADH5 | "alcohol dehydrogenase 5 (class III), chi polypeptide [Source:HGNC Symbol;Acc:HGNC:253]" | 4720 | 0.094 | 0.3416 | No |
| 18 | ALDH6A1 | aldehyde dehydrogenase 6 family member A1 [Source:HGNC Symbol;Acc:HGNC:7179] | 5560 | 0.028 | 0.2792 | No |
| 19 | ALDH3B2 | aldehyde dehydrogenase 3 family member B2 [Source:HGNC Symbol;Acc:HGNC:411] | 5617 | 0.022 | 0.2771 | No |
| 20 | DLAT | dihydrolipoamide S-acetyltransferase [Source:HGNC Symbol;Acc:HGNC:2896] | 5647 | 0.020 | 0.2769 | No |
| 21 | ALDH1A3 | aldehyde dehydrogenase 1 family member A3 [Source:HGNC Symbol;Acc:HGNC:409] | 5831 | 0.007 | 0.2633 | No |
| 22 | AKR1C4 | aldo-keto reductase family 1 member C4 [Source:HGNC Symbol;Acc:HGNC:387] | 5843 | 0.005 | 0.2630 | No |
| 23 | ALDH3B1 | aldehyde dehydrogenase 3 family member B1 [Source:HGNC Symbol;Acc:HGNC:410] | 5862 | 0.004 | 0.2621 | No |
| 24 | ALDH3A1 | aldehyde dehydrogenase 3 family member A1 [Source:HGNC Symbol;Acc:HGNC:405] | 6413 | -0.036 | 0.2230 | No |
| 25 | ALDH1B1 | aldehyde dehydrogenase 1 family member B1 [Source:HGNC Symbol;Acc:HGNC:407] | 6551 | -0.046 | 0.2170 | No |
| 26 | PDHX | pyruvate dehydrogenase complex component X [Source:HGNC Symbol;Acc:HGNC:21350] | 6760 | -0.060 | 0.2069 | No |
| 27 | ALDH8A1 | aldehyde dehydrogenase 8 family member A1 [Source:HGNC Symbol;Acc:HGNC:15471] | 7998 | -0.155 | 0.1264 | No |
| 28 | AKR1B10 | aldo-keto reductase family 1 member B10 [Source:HGNC Symbol;Acc:HGNC:382] | 8420 | -0.187 | 0.1126 | No |
| 29 | OGDH | oxoglutarate dehydrogenase [Source:HGNC Symbol;Acc:HGNC:8124] | 8503 | -0.194 | 0.1259 | No |
| 30 | PDHA2 | pyruvate dehydrogenase E1 subunit alpha 2 [Source:HGNC Symbol;Acc:HGNC:8807] | 9124 | -0.241 | 0.1022 | No |
| 31 | ALDH1L1 | aldehyde dehydrogenase 1 family member L1 [Source:HGNC Symbol;Acc:HGNC:3978] | 9302 | -0.256 | 0.1143 | No |
| 32 | GAPDH | glyceraldehyde-3-phosphate dehydrogenase [Source:HGNC Symbol;Acc:HGNC:4141] | 11268 | -0.467 | 0.0089 | No |
| 33 | ALDH1A2 | aldehyde dehydrogenase 1 family member A2 [Source:HGNC Symbol;Acc:HGNC:15472] | 11796 | -0.548 | 0.0235 | No |
| 34 | ALDH4A1 | aldehyde dehydrogenase 4 family member A1 [Source:HGNC Symbol;Acc:HGNC:406] | 12080 | -0.614 | 0.0637 | No |
Table: GSEA details [plain text format]

  

Fig 2: GOMF\_OXIDOREDUCTASE\_ACTIVITY\_ACTING\_ON\_THE\_ALDEHYDE\_OR\_OXO\_GROUP\_OF\_DONORS\_NAD\_OR\_NADP\_AS\_ACCEPTOR      
 Blue-Pink O' Gram in the Space of the Analyzed GeneSet

  

Fig 3: GOMF\_OXIDOREDUCTASE\_ACTIVITY\_ACTING\_ON\_THE\_ALDEHYDE\_OR\_OXO\_GROUP\_OF\_DONORS\_NAD\_OR\_NADP\_AS\_ACCEPTOR: Random ES distribution      
 Gene set null distribution of ES for **GOMF\_OXIDOREDUCTASE\_ACTIVITY\_ACTING\_ON\_THE\_ALDEHYDE\_OR\_OXO\_GROUP\_OF\_DONORS\_NAD\_OR\_NADP\_AS\_ACCEPTOR**
